# Basics and Best Practices of Multimodal Pain Management for the Plastic Surgeon

**DOI:** 10.1097/GOX.0000000000002833

**Published:** 2020-05-26

**Authors:** Jenny C. Barker, Girish P. Joshi, Jeffrey E. Janis

**Affiliations:** From the * Department of Plastic and Reconstructive Surgery, The Ohio State University Wexner Medical Center, Columbus, Ohio; † Center for Regenerative Medicine, Abigail Wexner Research Institute, Nationwide Children’s Hospital, Columbus, Ohio; ‡ Department of Anesthesiology and Pain Management, The University of Texas Southwestern Medical Center, Dallas, Tex.

## Abstract

Pain management is a central focus for the plastic surgeon’s perioperative planning, and it no longer represents a postoperative afterthought. Protocols that rely on opioid-only pain therapy are outdated and discouraged, as they do not achieve optimal pain relief, increase postoperative morbidity, and contribute to the growing opioid epidemic. A multimodal approach to pain management using non-opioid analgesic techniques is an integral component of enhanced recovery after surgery protocols. Careful perioperative planning for optimal pain management must be achieved in multidisciplinary collaboration with the perioperative care team including anesthesiology. This allows pain management interventions to occur at 3 critical opportunities—preoperative, intraoperative, and postoperative settings.

## INTRODUCTION

Optimal pain management is an essential component of enhanced recovery after surgery protocols (ERAS) that are becoming standard of care because they have been shown to reduce postoperative complications and expedite recovery.^1,2^ However, postoperative pain is still inadequately managed. Opioids remain the mainstay of perioperative pain management, despite well-recognized adverse events including nausea, vomiting, pruritus, sedation, constipation, respiratory depression, and opioid-induced hyperalgesia.^2,3^ In addition, opioid misuse and abuse has reached epidemic status. The surgeon’s contribution to the opioid epidemic is an undeniable reality. While surgeons are responsible for 10% of all opioid prescriptions,^4^ the most common reason for new opioid prescriptions is for acute postoperative pain.^5^ In 2017, the Commission on Combating Drug Addiction and the Opioid Crisis reported that “with approximately 142 Americans dying every day [from the opioid crisis], America is enduring a death toll equal to September 11th every three weeks.^6^” According to the US Center for Disease Control, 130 deaths per day are attributable to the opioid epidemic.^7^ In 2017, among 70,237 drug overdose deaths reported in 50 states, 47,600 (67.8%) involved an opioid.^8^ Two focused areas of concern describe potential surgeon contribution to the problem. The first is the creation of chronic opioid addiction in postsurgical patients who were previously opioid naive, termed “new persistent use.” For patients undergoing plastic surgery, specifically, the rates of new persistent use have been reproducibly demonstrated to be between 5% and 13% across a variety of plastic surgery procedures.^9,10^ For surgeons operating on pediatric patients, it is also important to note that the pediatric population is not immune to the development of new persistent use, with rates between 2% and 15% depending on the operation.^11,12^ Second, overprescribing by surgeons results in the inadvertent distribution of unused opioids into the community. Overprescribing increases the risk of “diversion” or the redirection of legally obtained prescription opioids for illicit abuse.^13,14^ Plastic surgeons are uniquely positioned to play an important role in the reduction of the opioid epidemic because of the efficacy of multimodal analgesia and ERAS protocols for plastic surgery procedures and because of the ability to influence multispecialty surgical collaboration.^15^ Multimodal analgesia is particularly applicable and effective for plastic surgery procedures, and the most prevalent strategies are reviewed herein.

## EVIDENCE-BASED STRATEGIES FOR EFFECTIVE PAIN MANAGEMENT

Multimodal analgesia is a strategy that reduces reliance on opioids through the use of non-opioid analgesics that have different mechanisms of action.^16,17^ Multimodal analgesia is directed toward 4 goals: (1) improvement in the patient experience through better pain control, (2) reduction in postoperative morbidity and mortality, (3) reduction in healthcare costs, and (4) decreased physician-contribution to the global opioid epidemic.

**Fig. 1.**
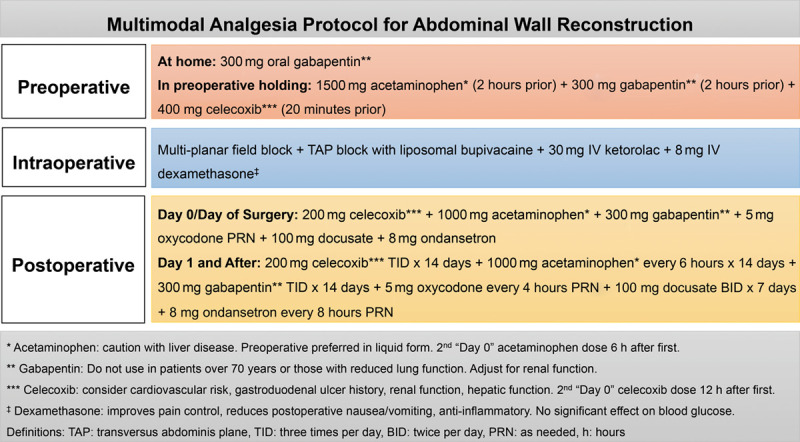
Multimodal analgesia protocol for abdominal wall reconstruction.

Multimodal analgesia regimens are both patient- and procedure-specific and utilize varying combinations of local and/or regional analgesic techniques and non-opioid analgesics (eg, acetaminophen, nonsteroidal anti-inflammatory drugs [NSAIDs], cyclooxygenase [COX]-2–specific inhibitors) and analgesic adjuncts (eg, dexamethasone, gabapentinoids). It is recommended that all surgical patients should receive “basic analgesics”, which include acetaminophen and either an NSAID or a COX-2-specific inhibitor unless there is a contraindication in a “scheduled” manner, perioperatively, preferentially over “as needed” or pro re nata administration.^2,18^ In addition, patients should receive some form of local/regional anesthetic technique (eg, surgical site infiltration, interfascial plane block). Furthermore, it is necessary to balance the invasiveness of the analgesic technique with the expected severity of postoperative pain and balance the efficacy of the analgesic technique and the potential adverse effects including the influence on ambulation.^2,18^ An example of how to incorporate these principles is presented in a sample multimodal analgesia protocol for abdominal wall reconstruction (Fig. 1).

## LOCAL AND REGIONAL ANALGESIC TECHNIQUES

With rare exception, local and/or regional anesthesia can and should be used as a matter of routine for most plastic surgery procedures. Local anesthetic medications inhibit neuronal action potentials via voltage-gated sodium channel blockade. The mode of administration can occur via single injection techniques or by catheter-based infusion techniques that administer local anesthetic either intermittently or continuously. Special consideration must be paid toward refining delivery techniques for local anesthetic administration to optimize patient comfort, especially for awake procedures.^19^

Evidence exists supporting the role of local anesthetics for opioid reduction, decreased postoperative nausea and vomiting (PONV), decreased pain scores, decreased lengths of stay, decreased rates of certain postoperative complications, and decreased healthcare costs across a variety of plastic surgery procedures.^20,21^ Therefore, it is imperative that the surgeon be familiar with anatomy and injection techniques and engage in a multidisciplinary approach to maximize the number of patients receiving regional anesthesia when clinically indicated.^22^ Joshi et al^22^ highlight that the persistent underuse of peripheral nerve blocks can be at least partially overcome by surgeon familiarity and administration of intraoperative interfascial plane blocks and appropriately performed surgical site infiltration. Technical considerations for surgical site infiltration as well as interfascial plane blocks have been recently reviewed.^23,24^

The challenge of short duration of action for most local anesthetics (approximately 8–12 hours) has been met by catheter infusion techniques or through the development of extended release formulations such as liposomal bupivacaine. In a systematic review of 28 publications encompassing plastic surgery procedures, catheter-based infusion of local anesthetic resulted in an opioid-sparing effect in 92% of studies reviewed.^25^

Recent evidence has emerged highlighting the benefit of extended release formulations due to prolonged duration of analgesia, ease of single-injection technique that does not necessitate catheter placement, and long-term cost-effectiveness. A 2016 meta-analysis demonstrated safety, tolerability, and either comparable or increased efficacy for pain control for the use of liposomal bupivacaine in plastic surgery.^16^ Liposomal bupivacaine for regional blockade in the transversus abdominis plane is commonly used in abdominal procedures such as abdominally based microsurgical breast reconstruction and abdominal wall reconstruction,^26^ demonstrating opioid reduction, decreased lengths of stay, and improved pain scores^27–31^ over traditional catheter-based infusion techniques^28,29^ without increases in wound complications.^32^ Studies have demonstrated either equivalent^27,28^ or long-term cost-effectiveness of liposomal bupivacaine including decreased length of stay and lower 30-day readmission rates.^33^

It is not only critical for the plastic surgeon to become familiar with techniques of local and regional anesthesia but also frequently refresh knowledge of the management of complications associated with these medications.^34^ Moreover, frontline providers in both the inpatient and office setting must be educated on signs of local anesthetic toxicity, with protocols in place for management.^22^

### Recommendations Summary

Use of local and/or regional anesthesia is highly recommended. For many plastic surgery procedures, single regional injection of extended release formulations has the advantage of long duration of action without the need for catheter placement and management. Surgeons must become familiar with regional anatomy and techniques and implement protocols including refresher education for frontline providers on symptoms and management of local anesthetic toxicity.^22^

## NSAIDS AND COX-2-SPECIFIC INHIBITORS

NSAIDs form the basis of an optimal multimodal analgesic technique. For core plastic surgery procedures, the use of celecoxib has been shown to reduce pain scores, reduce opioid consumption, expedite return of bowel function and return to activities of daily living, and improve patient satisfaction.^35^ In outpatient breast plastic surgery, the use of NSAIDs within a preoperative multimodal analgesia protocol reduced opioid requirements in the postanesthesia care unit by 40% and improved pain scores by 40%.^36^ NSAIDs are more effective than other adjunct oral medications with lower numbers needed to treat.^37^

COX-2 selective inhibitors have a more favorable side effect profile with regard to bleeding than nonselective modalities but are contraindicated in those with preexisting coronary artery disease because of their association with higher rates of cardiac events. NSAIDs and COX-2 selective inhibitors should also be avoided in those with acute or chronic renal disease, and iatrogenic acute renal injury is also a consideration for older perioperative patients, though its absolute risk is not well-defined for plastic surgery procedures.^38^ Controversy surrounds the risk of impaired platelet function and potential surgical bleeding risk associated with NSAIDs, specifically as it relates to reduction mammoplasty.^39^ However, there remains no high-level evidence to support their contraindication in most plastic surgical patients, and several large reviews have concluded that there is no difference in hematoma rates associated with NSAID use.^37,40–42^ A recent meta-analysis reviewing 15 studies with 3064 cumulative patients demonstrated no increase in hematoma with the use of several types of NSAIDs across a variety of plastic surgical procedures including breast surgery.^43^ In breast and body contouring procedures, Kelley et al reviewed 106,279 patients; among these, 4,924 (4.6%) patients received postoperative ketorolac.^44^ Multivariable regression analysis demonstrated that ketorolac was not associated with hematoma (odds ratio, 1.20; 95% CI, 0.99–1.46; *P* > 0.05), concluding that the benefits of NSAID use outweigh the risks. In sum, the anecdotal frequency of hematoma formation secondary to NSAIDs is overestimated, with evidence supporting the use of NSAIDs in the appropriately selected patient.

### Recommendations Summary

The benefits of NSAID or COX-2 selective inhibitor use in the appropriately selected patient outweigh the risks. Preoperative, intraoperative, and postoperative incorporation of these analgesics into multimodal analgesia regimens is recommended.

## ACETAMINOPHEN

Acetaminophen is an effective perioperative analgesic with efficacy for both pain control and opioid reduction across all surgical specialties.^45^ The mechanism of action is unknown, although is thought to be from activation of descending serotonergic pathways and inhibition of COX-mediated prostaglandin synthesis in the central nervous system.^46^ The safety profile of acetaminophen is favorable, with the exception of hepatotoxicity above the maximum dosage of 4 g per 24 hours. Avoiding acetaminophen usage in patients with known liver disease should be considered. Controversy has centered around the effectiveness of oral versus parenteral formulations and its relation to cost. A recent meta-analysis demonstrates no clear advantage of parenteral administration over oral administration for appropriately selected patients for the indication of perioperative pain control.^47^ Oral administration has been reproducibly demonstrated to be effective for preoperative and postoperative analgesia, specifically.^36,48,49^ Oral dosage protocols range from 650 to 1000 mg and favor “around-the-clock” administration, typically every 6 hours. There have been no high-level studies comparing the effectiveness of dosage differences for the indication of perioperative pain management. With “around-the-clock” acetaminophen regimens, it is recommended to avoid the use of acetaminophen–opioid combination formulations for breakthrough pain because of the risk of inadvertent acetaminophen overdose. This warrants special mention because combination formulations are the most commonly prescribed by plastic surgeons for breakthrough pain.^50^

### Recommendations Summary

Acetaminophen should be used routinely for preoperative, intraoperative, and postoperative analgesia in an “around-the-clock” manner to reduce pain and opioid consumption. It is not recommended to prescribe acetaminophen–opioid combination tablets for breakthrough pain with this regimen because of the risk of acetaminophen overdose. Both oral and parenteral formulations are effective. Consideration should be given to avoid acetaminophen in patients with known liver disease.

## GABAPENTINOIDS

Gabapentin is the most widely used anticonvulsant as an adjunct perioperative pain medication. In the perioperative setting, gabapentin decreases postoperative pain and opioid consumption, as well as PONV.^36,51,52^ A meta-analysis encompassing 18 studies and 1,181 patients demonstrated that perioperative gabapentin resulted in 35% reduction in total opioid consumption and pain within the first 24 hours after surgery while simultaneously increasing early ambulation.^53^ A large randomized controlled trial encompassing a variety of surgical procedures demonstrated effectiveness of perioperative gabapentin toward supporting postoperative opioid cessation, with a regimen consisting of 1,200 mg preoperatively and 600 mg 3 times daily postoperatively for 10 doses.^54^ In the burn population, gabapentin is uniquely beneficial because of its ability to reduce burn-associated pruritus, in addition to decreasing opioid consumption.^55,56^

Of note, most studies assessing the analgesic benefits of gabapentinoids did not use the basic analgesics (ie, acetaminophen and NSAIDs) or local anesthetic techniques. The benefits of gabapentinoids over basic analgesic techniques appear to be limited.^1,2^ Furthermore, gabapentinoids are associated with several adverse effects including dizziness, somnolence, and respiratory depression. For this reason, gabapentinoids should be used selectively when basic analgesic techniques are not possible or contraindicated and patients are at a high risk of persistent postoperative pain.^1,2^ Also, it is recommended to avoid or dose-reduce the medication for the elderly, patients with significant comorbidities, and those with reduced lung function including chronic obstructive pulmonary disease or known or suspected obstructive sleep apnea.^57^

### Recommendations Summary

Gabapentin is a useful analgesic adjunct in the preoperative and postoperative setting that can reduce opioid consumption and improve the patient’s postoperative experience. However, gabapentin should be used in selective patient populations and surgical procedures that are at a high risk of persistent postoperative pain. In these situations, gabapentin should not be avoided because of physician lack of familiarity with the drug. Concerns with gabapentinoids include dizziness and sedation, as well as potential for respiratory depression, particularly in the patients with significant comorbidities, the elderly, and patients with known or suspected reduced lung function such as chronic obstructive pulmonary disease or obstructive sleep apnea.

## DEXAMETHASONE

Glucocorticoids reduce postoperative pain through anti-inflammatory mechanisms and possess antiemetic properties, serving as a key adjunct for PONV prophylaxis.^58^ A 2013 meta-analysis demonstrated that postoperative patients have decreased pain scores and opioid consumption when dexamethasone is included as a multimodal adjunct.^59^ Few studies address the role of dexamethasone in plastic surgery, specifically. A recent prospective, double-blind randomized trial in head and neck microvascular reconstruction similarly demonstrated decreased pain and opioid consumption after dexamethasone inclusion. However, hyperglycemia and insulin requirements were problematic for patients in the treatment group, leading the authors to conclude that the risks for reconstructive patients outweighed the benefits.^60^ In contrast, systematic reviews have concluded that the benefits from dexamethasone outweigh the concerns of hyperglycemia, except for patients with brittle diabetes mellitus. No evidence currently exists correlating dexamethasone use with an increased occurrence of postoperative wound infection or wound healing delay.^61^

### Recommendations Summary

Dexamethasone reduces pain and opioid consumption and reduces PONV. A single intraoperative dose of dexamethasone 8 mg, IV should be considered as an integral component of multimodal analgesia and antiemetic prophylaxis for all procedures unless there is a contraindication (eg, uncontrolled diabetes mellitus). Those at risk for complications associated with postoperative hyperglycemia represent less ideal candidates.

## WHAT PATIENTS SHOULD KNOW

Early communication, establishment of expectations, and patient engagement are necessary for multimodal analgesia to be most effective. In 2017, the final report from The President’s Commission on Combating Drug Addiction and the Opioid Crisis cited the lack of patient education as a significant contributor to the current epidemic. Specially, that “patients and their families are not often fully informed regarding whether their prescriptions are opioids, the risks of opioid addiction or overdose, control and diversion, dose escalation, or the risks of use with alcohol or benzodiazepines.”^61^ For plastic surgery patients, specific points that should routinely be communicated to patients in the preoperative clinic setting include (1) discussion of the medical risks of opioids, in terms of known postoperative side effects and potential for abuse, (2) purpose and importance of alternative medications and regional procedures, and (3) proper medication storage and proper disposal (Table 1).

**Table 1.** Recommended Counseling Points Regarding Multimodal Analgesia and Appropriate Use of Opioid Medications for Plastic Surgery Patients and Families

Topic	Recommended Details
Opioid-related adverse events	Common side effects are nausea, vomiting, constipation, pruritus, sedation, dizziness. Pain regimens based on opioids increase the risk of respiratory depression, venous thromboembolism, postoperative infections, longer length of stay, and increased costs.
New persistent use and opioid addiction	5%–13% of previously opioid naive plastic surgery patients develop new persistent use.
Opioids for breakthrough pain only	Smaller prescription sizes do not diminish quality of pain control when used in conjunction with multimodal analgesia.
Benefits of regional analgesia	Discuss what to expect when, so that patients are well informed before any preoperative procedures.
Purpose of adjunct multimodal analgesia pain medications	Empower patients with drug name, class, dose, duration, and evidence-based benefits.
Proper opioid storage in a locked location	Most instances of diversion are through family, friends, and acquaintances. “It can happen to anyone.”
Disposal of opioids	Rediscuss diversion. Patient education materials available are on the CDC website for disposal options.

Counseling should occur in the preoperative setting and be reiterated postoperatively.

Education regarding proper storage and disposal are linked to risk minimization for opioid diversion. A recent meta-analysis of 7 different surgical procedures demonstrated that 42%–71% of opioid tablets that are prescribed are not used after surgery.^62^ In a study of secondary breast procedures and breast reduction, patients reported an average of 12–18 leftover tablets per operation.^63^ Further, over 70% of postoperative prescription opioids are not safely stored in a locked location.^62^ The majority of patients do not know how to safely dispose of unused medication. This prevalence of overprescribed, excess tablets is problematic but provides an opportunity for intervention. Interestingly, the simple act of providing smaller prescriptions has been shown to decrease opioid consumption without impacting quality of pain control.^64^ A recent prospective trial demonstrated that preoperative education results in 20% reduction of opioid consumption.^64^ Likewise, patient education has also been shown to increase the frequency of proper disposal by 22%.^65^ Unfortunately, a recent review of prescribing practices in plastic surgery notes that only 45% and 37% of surgeons report routinely counseling their patients on the risks of addiction and secure medication storage, respectively.^48^

### Recommendations Summary

Patients should be informed that overprescribing does not improve quality of pain control. It is imperative to engage patients early to communicate known adverse effects associated with opioid use. Further, patients should be counseled to expect multiple opportunities for alternative pain management interventions throughout their preoperative, intraoperative, and postoperative course. Counseling regarding proper medication storage and disposal is the responsibility of every prescription provider.

## CONCLUSIONS

Multimodal analgesia with non-opioid analgesics and local anesthetic techniques should be incorporated into every surgical procedure, with opioids prescribed only for rescue analgesia (ie, for breakthrough pain) and not as a primary pain management strategy. While direct comparisons between protocols do not exist for a majority of plastic surgery operations, principles of multimodal analgesia carry through (Table 2). It is important to note that interventions should occur across 3 time points in the preoperative, intraoperative, and postoperative settings and in multidisciplinary collaboration. Standardization of protocols increases efficiency and compliance across a healthcare system and is also known to decrease cost.^66^ Therefore, it is recommended to develop procedure-specific protocols for one’s practice that are well-communicated with patients, families, and collaborative practitioners. Although outside the scope of this article, it is critical to identify patients at a higher risk of postoperative pain and emphasize that approach to pain therapy based exclusively on pain scores is inappropriate.

**Table 2.** Recommendations Summary for Principles of Multimodal Analgesia

Multimodal Agent	Useful Indications and Timing	Important Considerations and Contraindications
Local and regional anesthetic	All patients should receive local or regional anesthetic	Balance invasiveness of technique with severity of pain
		Refresh providers on signs/symptoms of local anesthetic toxicity
Nonsteroidal anti-inflammatory drugs and COX-2 selective inhibitors	Recommended for all patients unless contraindicated	Contraindicated in chronic or acute kidney injury
	Effective in “scheduled” manner perioperatively	COX-2 inhibitors should be avoided in patients with coronary artery disease
	Can be implemented in preoperative, intraoperative, and postoperative settings	Does not increase bleeding complications
Acetaminophen	Recommended for all patients unless contraindicated	Do not exceed 4 g in 24 h
	Effective in “scheduled” manner perioperatively	Use caution in patients with known liver disease
	Can be implemented in preoperative, intraoperative, and postoperative settings	It is not recommended to prescribe combination opioid-acetaminophen medications with a scheduled acetaminophen regimen to avoid inadvertent toxicity
Gabapentinoids	Useful for operations at higher risk for persistent postoperative pain	Adverse events include dizziness, somnolence, and decreased respiration
		Caution advised with elderly patients and those with reduced lung function
	Can be implemented in preoperative and postoperative settings.	Dose-adjusted for renal function
Dexamethasone	Single intraoperative dose useful for analgesia and antiemetic prophylaxis	Monitor for perioperative hyperglycemia, but no evidence to strongly support this as a contraindication
	Consider especially for patients with history of PONV	

## ACKNOWLEDGMENTS

J.B. was supported by the National Institutes of Health (NIH) T32AI106704-01A1, NIH F32HL144120-01A1, and the Ohio State University President’s Postdoctoral Scholar Program.
